# Predictive value of nutritional biomarkers for immune checkpoint inhibitor efficacy in patients with non-small cell lung cancer

**DOI:** 10.3389/fnut.2026.1882630

**Published:** 2026-08-03

**Authors:** Wentao Zhang, Xia Liu

**Affiliations:** 1 Department of Gastrointestinal Surgery, Southeast University Affiliated Xuzhou Central Hospital, Xuzhou, China; 2 Department of Respiratory and Critical Care Medicine, Southeast University Affiliated Xuzhou Central Hospital, Xuzhou, China

**Keywords:** albumin, body mass index, immune checkpoint inhibitors, non-small cell lung cancer, prediction, prognostic nutritional index

## Abstract

**Background:**

Although immune checkpoint inhibitors (ICIs) have revolutionized advanced non-small cell lung cancer (NSCLC) treatment, clinical benefits vary. This study evaluated the predictive value of baseline nutritional and hematological markers for ICI efficacy and survival.

**Methods:**

A retrospective cohort of 124 advanced NSCLC patients receiving ICIs was evaluated. Patients were stratified by median cut-offs: prognostic nutritional index (PNI: 45), albumin (ALB: 40.2 g/L), body mass index (BMI: 20.1 kg/m^2^), and hemoglobin (Hb: 114 g/L). Independent predictors of binary short-term efficacy outcomes (ORR and DCR) and time-to-event long-term survival outcomes (PFS and OS) were identified using multivariate logistic regression and Cox proportional hazards regression models.

**Results:**

High baseline PNI and ALB were significantly associated with superior objective response and disease control rates (DCR). Multivariate analyses confirmed PNI (OR = 1.377, *p* = 0.014) and ALB (OR = 1.840, *p* = 0.003) as independent protective factors for DCR, alongside independent associations with prolonged progression-free and overall survival (OS) (all *p* < 0.05). BMI lost independent predictive value after multivariate adjustment. The combined PNI + ALB model consistently outperformed single markers, achieving 12-, 24-, and 36-month OS area under the curve (AUC) values of 0.802, 0.819, and 0.807, respectively.

**Conclusion:**

Baseline PNI and ALB are non-invasive, independent predictors of short-term response and long-term survival in advanced NSCLC patients undergoing immunotherapy. Combining these markers improves prognostic discrimination.

## 1 Introduction

Non-small cell lung cancer (NSCLC) is one of the most common malignancies worldwide with high morbidity and mortality, accounting for approximately 85% of all lung cancer cases (1). Despite significant advances in surgery, radiotherapy, and targeted therapy in recent years, the prognosis for patients with advanced (stage IIIB–IV) NSCLC remains poor (2). Immune checkpoint inhibitors (ICIs), particularly those targeting programmed death-1 (PD-1) and its ligand (PD-L1), have fundamentally changed the treatment landscape for advanced NSCLC (3). Multiple phase III randomized controlled trials (e.g., KEYNOTE series, CheckMate series) have demonstrated that ICI monotherapy or combination with chemotherapy significantly prolongs progression-free survival (PFS) and overall survival (OS) in advanced NSCLC patients, with some patients achieving durable remission (4). However, the clinical benefit of immunotherapy is not uniformly distributed across all NSCLC patients receiving ICIs. Large-scale clinical data indicate that only about 20–40% of patients show objective response to ICI therapy, while the majority may develop primary or acquired resistance (5). Therefore, identifying reliable biomarkers to predict immunotherapy efficacy and enable precise individualized treatment selection has become a central research focus in oncology (6).

Currently, the most widely used predictive biomarker for immunotherapy in clinical practice is tumor cell PD-L1 expression level (7). In addition, tumor mutational burden (TMB), microsatellite instability (MSI), tumor-infiltrating lymphocytes (TILs), and circulating tumor DNA (ctDNA) have also been suggested to have varying degrees of predictive value (8). However, these biomarkers have obvious limitations in clinical application. PD-L1 expression is significantly influenced by detection methods, antibody clones, and scoring criteria, leading to poor consistency across different testing platforms (9). TMB detection relies on next-generation sequencing technology, which is costly and lacks standardization (10). Moreover, most of these molecular markers require tissue sample testing or expensive molecular experiments, limiting their accessibility and convenience in routine clinical practice (11).

Immuno-nutritional markers based on peripheral blood or routine hematological tests have gradually attracted attention due to their advantages of non-invasiveness, low cost, and good reproducibility (12). There is a close interaction between the body’s nutritional status and immune function. Malnutrition can impair the host’s anti-tumor immune response through various mechanisms, including but not limited to reduced number and functional decline of T cell subsets, decreased natural killer (NK) cell activity, and abnormal regulation of immune checkpoint molecule expression (13). The prognostic nutritional index (PNI) is a composite marker that reflects both immune and nutritional status, calculated from serum albumin (ALB) levels and peripheral blood lymphocyte count (14). Numerous studies have confirmed that PNI has significant prognostic value in various solid tumors (including gastric, colorectal, and liver cancers) (15). In recent years, the application of PNI in predicting ICI therapy efficacy in NSCLC patients has gradually increased. Multiple retrospective studies and meta-analyses have shown that higher baseline PNI levels are significantly associated with better objective response rate (ORR), longer PFS, and longer OS in NSCLC patients receiving ICIs (16, 17). Serum albumin (ALB), an important indicator of nutritional status and inflammatory response, has been widely used in prognostic assessment of cancer patients. Hypoalbuminemia is often closely associated with increased systemic inflammation, reduced immune function, and tumor consumption (18). Body mass index (BMI), a simple height-weight assessment tool, has also shown growing research value in the field of cancer immunotherapy. The obesity paradox suggests that among immunotherapy populations, overweight or mildly obese patients may achieve better treatment outcomes, possibly related to the immunomodulatory functions of adipose tissue and obesity-related chronic low-grade inflammation (19). Hemoglobin (Hb), a primary screening indicator for anemia, is closely related to performance status and quality of life in cancer patients, but its predictive value for ICI therapy efficacy remains controversial (20).

Although existing studies have preliminarily confirmed the predictive potential of these nutritional markers in ICI therapy, several limitations remain. First, most studies focus only on the prognostic value of a single marker, lacking systematic evaluation of the combined predictive efficacy of multiple nutritional markers. Second, there is heterogeneity in the definition and cut-off value selection of nutritional markers across studies, limiting standardization for clinical application. Finally, evidence regarding the relationship between baseline nutritional characteristics and ICI efficacy in the Chinese population is still relatively limited. Based on this, our study systematically analyzed the predictive value of four baseline routine nutritional and hematological markers—PNI, ALB, BMI, and Hb—for short-term efficacy (ORR and DCR) and long-term survival (PFS and OS) in advanced NSCLC patients receiving ICI therapy, and further constructed a multi-marker combined prediction model to evaluate its clinical discriminative ability. This study aims to provide new evidence for precision stratification and individualized intervention in immunotherapy for advanced NSCLC.

## 2 Methods

### 2.1 Study population

This retrospective cohort study consecutively included patients with advanced non-small cell lung cancer (NSCLC) who received immune checkpoint inhibitor (ICI) therapy at the Department of Oncology of a tertiary hospital from January 2021 to February 2026. Inclusion criteria were as follows: (1) pathologically confirmed NSCLC with clinical stage IIIB–IV (according to the American Joint Committee on Cancer 8th edition TNM staging) or postoperative recurrence/metastasis; (2) received PD-1/PD-L1 inhibitor monotherapy or combination with chemotherapy as first-line or later-line treatment, with at least 2 treatment cycles completed; (3) had complete baseline data within 14 days before the first ICI treatment, including complete blood count, serum albumin (ALB), lactate dehydrogenase (LDH), and body mass index (BMI); (4) at least one imaging evaluation of efficacy completed. Exclusion criteria included: (1) active infection before treatment; (2) concomitant autoimmune disease; (3) long-term (≥2 weeks) use of glucocorticoids (equivalent prednisone >10 mg/d) or other immunosuppressants; (4) other concurrent malignancies; (5) severely missing baseline or follow-up data. This study was approved by the Ethics Committee of Xuzhou Central Hospital (ethics approval number: XZXY-LJ-20260510-091).

### 2.2 Clinical data collection and grouping methods

Baseline demographic and clinical characteristics were collected, including gender, age (<60 years vs. ≥60 years), smoking history, histological subtype (squamous cell carcinoma, adenocarcinoma, others), Eastern Cooperative Oncology Group (ECOG) performance status (0–1 vs. 2), and treatment regimen (monotherapy vs. combination therapy). PNI, ALB, BMI, and Hb were obtained from the most recent clinical measurements or records prior to initiation of immune checkpoint inhibitor therapy. Serum albumin and lymphocyte counts were derived from routine biochemical and complete blood count examinations performed during the same period. Patients with acute infection, missing data, or incomplete baseline assessments were excluded according to predefined inclusion and exclusion criteria. The median value of each indicator was used as the cut-off value to divide patients into high-level and low-level groups in order to maximize statistical power and avoid arbitrary investigator bias, a pragmatic approach frequently adopted in exploratory retrospective cohorts. The PNI was calculated using the following standard formula: PNI = serum albumin level (g/L) + 5 × total lymphocyte count (10^9/L). The specific grouping criteria were as follows: PNI cut-off value of 45, Hb cut-off value of 114 g/L, ALB cut-off value of 40.2 g/L, and BMI cut-off value of 20.1 kg/m^2^.

### 2.3 Treatment regimens

All patients received programmed death-1 (PD-1) or programmed death-ligand 1 (PD-L1) inhibitor monotherapy or combination therapy. Specific immunotherapeutic agents used included camrelizumab, pembrolizumab, sintilimab, and tislelizumab. Combination therapy included platinum-based doublet chemotherapy. Efficacy was evaluated every 2–3 cycles, and treatment continued until disease progression or unacceptable toxicity.

### 2.4 Efficacy and survival outcome assessment

Tumor response was classified as complete response (CR), partial response (PR), stable disease (SD), and progressive disease (PD). Objective response rate (ORR) was defined as (CR + PR)/total number × 100%; disease control rate (DCR) was defined as (CR + PR + SD)/total number × 100%. Survival outcomes included progression-free survival (PFS) and overall survival (OS). PFS was defined as the time from the first ICI treatment to first radiographic progression or death from any cause (whichever occurred first). OS was defined as the time from the first ICI treatment to death from any cause or last follow-up.

### 2.5 Statistical analysis

All statistical analyses were performed using SPSS 26.0 and R software (version 4.2.2). Normally distributed continuous variables were expressed as mean ± standard deviation, while non-normally distributed continuous variables were expressed as median and interquartile range; categorical variables were expressed as number (frequency) and percentage. Comparisons of continuous variables between two groups were performed using independent sample *t*-test or Mann–Whitney *U* test, and categorical variables were compared using the *χ*^2^ test; comparisons among multiple groups were performed using analysis of variance or Kruskal–Wallis *H* test.

Univariate and multivariate binary logistic regression models were used to evaluate the independent predictive value of PNI, ALB, BMI, and other indicators for short-term efficacy (ORR and DCR), calculating odds ratios (OR) and their 95% confidence intervals (CI). Univariate and multivariate Cox proportional hazards regression models were used to identify prognostic factors affecting OS and PFS. The results are presented as hazard ratio (HR) with corresponding 95% confidence intervals (95% CIs) and *p* values. Kaplan–Meier curves (using R packages survival and survminer) were plotted to display survival distributions, and the log-rank test was used to compare survival differences between groups. To further evaluate the predictive performance of PNI, ALB, and their combined model for OS and PFS, a multivariate Cox proportional hazards model was constructed, and the linear predictor derived from this model was used as the combined risk score. Time-dependent receiver operating characteristic (tROC) curve analysis was then performed to assess the prognostic performance of PNI, ALB, and the combined PNI + ALB model for 12-, 24-, and 36-month OS and PFS. The area under the curve (AUC) and its 95% confidence interval (95% CI) were calculated. Internal validation of the prognostic model was performed using bootstrap resampling with 1,000 repetitions to minimize overfitting and evaluate model stability. The model was estimated by repeatedly refitting the Cox proportional hazards model in bootstrap samples. Calibration performance was evaluated at 12, 24, and 36 months using bootstrap-corrected calibration curves. Predicted survival probabilities were derived from the Cox model, while observed probabilities were estimated using the Kaplan–Meier method. The calibration accuracy was assessed by the agreement between predicted and observed probabilities, with the 45-degree line representing perfect concordance. All statistical tests were two-sided, and *p* < 0.05 was considered statistically significant.

## 3 Results

### 3.1 Cohort selection and baseline clinical characteristics of patients

Following strict application of the inclusion and exclusion criteria, a total of 124 patients with advanced NSCLC who received ICI therapy at our hospital were finally included in this retrospective analysis cohort. Regarding baseline demographic characteristics, male patients were predominant, with 84 (67.7%) males and 40 (32.3%) females. The cohort showed a significant aging trend, with 91 (73.4%) patients aged ≥60 years and 33 (26.6%) aged <60 years. Regarding smoking history, 71.0% (88 patients) had a definite smoking history. In terms of tumor pathology and clinical characteristics, adenocarcinoma was the most common histological subtype, accounting for 71.8% (89 patients), while non-adenocarcinoma carcinoma accounted for 28.2% (35 patients). ECOG performance status was generally good, with 77.4% (96 patients) having a score of 0–1, indicating that most patients had good baseline physical function before receiving immunotherapy. Regarding treatment strategy, 64.5% (80 patients) received ICI-based combination therapy (e.g., immunotherapy plus chemotherapy), while the remaining 35.5% (44 patients) received ICI monotherapy.

To investigate the potential impact of baseline nutritional and hematological status on treatment, we calculated the specific values of PNI, ALB, BMI, and Hb before the first immunotherapy. The median values in the entire cohort were used as statistical cut-off values, which were: PNI = 45, ALB = 40.2 g/L, BMI = 20.1 kg/m^2^, and Hb = 114 g/L, allowing division of all patients into corresponding high-level and low-level groups. Subsequent Pearson chi-square (*χ*^2^) tests showed no statistically significant differences in the distribution of gender, age, smoking history, pathological type, ECOG score, or treatment regimen between the high- and low-level groups for each indicator (all *p* > 0.05). These results suggested that the major baseline characteristics were generally comparable between groups (Table 1).

**Table 1:** Baseline patient characteristics and grouping.

Characteristic	Total (*N* = 124)	PNI < 45 (*N* = 61)	PNI ≥ 45 (*N* = 63)	*p* value	Hb < 114 (*N* = 58)	Hb ≥ 114 (*N* = 66)	*p* value	ALB < 40.2 (*N* = 55)	ALB ≥ 40.2 (*N* = 69)	*p* value	BMI < 20.1 (*N* = 52)	BMI ≥ 20.1 (*N* = 72)	*p* value
Gender				0.158			0.297			0.289			0.280
Male	84 (67.7)	45 (73.8)	39 (61.9)		42 (72.4)	42 (63.6)		40 (72.7)	44 (63.8)		38 (73.1)	46 (63.9)	
Female	40 (32.3)	16 (26.2)	24 (38.1)		16 (27.6)	24 (36.4)		15 (27.3)	25 (36.2)		14 (26.9)	26 (36.1)	
Age				0.473			0.524			0.577			0.632
<60	33 (26.6)	18 (29.5)	15 (23.8)		17 (29.3)	16 (24.2)		16 (29.1)	17 (24.6)		15 (28.8)	18 (25.0)	
≥60	91 (73.4)	43 (70.5)	48 (76.2)		41 (70.7)	50 (75.8)		39 (70.9)	52 (75.4)		37 (71.2)	54 (75.0)	
Smoking history				0.284			0.260			0.237			0.214
Yes	88 (71.0)	46 (75.4)	42 (66.7)		44 (75.9)	44 (66.7)		42 (76.4)	46 (66.7)		40 (76.9)	48 (66.7)	
No	36 (29.0)	15 (24.6)	21 (33.3)		14 (24.1)	22 (33.3)		13 (23.6)	23 (33.3)		12 (23.1)	24 (33.3)	
Histology				0.855			0.894			0.921			0.935
Adenocarcinoma	89 (71.8)	44 (72.1)	45 (71.4)		42 (72.4)	47 (71.2)		40 (72.7)	49 (71.0)		38 (73.1)	51 (70.8)	
Squamous	32 (25.8)	16 (26.2)	16 (25.4)		15 (25.9)	17 (25.8)		14 (25.5)	18 (26.1)		13 (25.0)	19 (26.4)	
Others	3 (2.4)	1 (1.6)	2 (3.2)		1 (1.7)	2 (3.0)		1 (1.8)	2 (2.9)		1 (1.9)	2 (2.8)	
ECOG				0.339			0.211			0.265			0.326
0–1	96 (77.4)	45 (73.8)	51 (81.0)		42 (72.4)	54 (81.8)		40 (72.7)	56 (81.2)		38 (73.1)	58 (80.6)	
2	28 (22.6)	16 (26.2)	12 (19.0)		16 (27.6)	12 (18.2)		15 (27.3)	13 (18.8)		14 (26.9)	14 (19.4)	
Treatment				0.377			0.363			0.348			0.332
Monotherapy	44 (35.5)	24 (39.3)	20 (31.7)		23 (39.7)	21 (31.8)		22 (40.0)	22 (31.9)		21 (40.4)	23 (31.9)	
Combination	80 (64.5)	37 (60.7)	43 (68.3)		35 (60.3)	45 (68.2)		33 (60.0)	47 (68.1)		31 (59.6)	49 (68.1)	

Data are presented as *n* (%). *p* values were calculated using the *χ*^2^ test. PNI, prognostic nutritional index; Hb, hemoglobin; ALB, albumin; BMI, body mass index; ECOG, Eastern Cooperative Oncology Group.

### 3.2 Association of nutritional Indicators with short-term efficacy and independent predictive analysis

Cross-tabulation and between-group comparisons clearly showed that patients with high baseline PNI and ALB levels had significantly better short-term tumor regression and disease stabilization after ICI therapy. Specifically, the ORR in the baseline PNI ≥ 45 group was 38.1%, significantly higher than that in the PNI < 45 group (19.7%, *p* = 0.024); meanwhile, the DCR reached 85.7%, which was highly significantly different from that in the low-level group (60.7%, *p* = 0.002). This significant advantage was also observed for ALB: the ALB ≥ 40.2 group had significantly higher ORR (37.7% vs. 18.2%, *p* = 0.017) and DCR (85.5% vs. 58.2%, *p* = 0.001) than the low albumin group. For BMI, the BMI ≥ 20.1 group showed a significantly higher ORR (37.5% vs. 17.3%, *p* = 0.015), although there was a numerical trend toward higher DCR, the difference between the two groups did not reach statistical significance (*p* = 0.153). In addition, no significant effect on short-term efficacy (ORR and DCR) was observed between different Hb level groups (both *p* > 0.05) (Table 2).

**Table 2:** Treatment response according to nutritional indicator groups.

Outcome	PNI < 45 (*N* = 61)	PNI ≥ 45 (*N* = 63)	*p* value	Hb < 114 (*N* = 58)	Hb ≥ 114 (*N* = 66)	*p* value	ALB < 40.2 (*N* = 55)	ALB ≥ 40.2 (*N* = 69)	*p* value	BMI < 20.1 (*N* = 52)	BMI ≥ 20.1 (*N* = 72)	*p* value
CR	0 (0.0%)	1 (1.6%)		1 (1.7%)	0 (0.0%)		0 (0.0%)	1 (1.4%)		0 (0.0%)	1 (1.4%)	
PR	12 (19.7%)	23 (36.5%)		11 (19.0%)	14 (21.2%)		10 (18.2%)	25 (36.2%)		9 (17.3%)	26 (36.1%)	
SD	25 (41.0%)	30 (47.6%)		34 (58.6%)	31 (47.0%)		22 (40.0%)	33 (47.8%)		23 (44.2%)	26 (36.1%)	
PD	24 (39.3%)	9 (14.3%)		13 (22.4%)	20 (30.3%)		23 (41.8%)	10 (14.5%)		20 (38.5%)	19 (26.4%)	
ORR	12 (19.7%)	24 (38.1%)	0.024	12 (20.7%)	14 (21.2%)	0.870	10 (18.2%)	26 (37.7%)	0.017	9 (17.3%)	27 (37.5%)	0.015
DCR	37 (60.7%)	54 (85.7%)	0.002	44 (75.9%)	45 (68.2%)	0.272	32 (58.2%)	59 (85.5%)	0.001	32 (61.5%)	53 (73.6%)	0.153

Data are presented as *n* (%). *p* values were calculated using the *χ*^2^ test. CR, complete response; PR, partial response; SD, stable disease; PD, progressive disease; ORR, objective response rate; DCR, disease control rate; PNI, prognostic nutritional index; Hb, hemoglobin; ALB, albumin; BMI, body mass index.

Univariate binary logistic regression analysis showed that higher baseline PNI, ALB, and BMI were significantly associated with better short-term treatment response. For ORR, PNI (OR = 1.207, 95% CI: 1.033–1.411, *p* = 0.018), ALB (OR = 1.188, 95% CI: 1.029–1.371, *p* = 0.019), and BMI (OR = 1.131, 95% CI: 1.030–1.243, *p* = 0.010) were all significantly associated with an increased likelihood of achieving objective response. Similarly, for DCR, PNI (OR = 1.325, 95% CI: 1.113–1.577, *p* = 0.002), ALB (OR = 1.500, 95% CI: 1.239–1.817, *p* < 0.001), and BMI (OR = 1.176, 95% CI: 1.047–1.321, *p* = 0.006) were significantly associated with improved disease control (Table 3). Variables that were significant in the univariate logistic regression analysis for DCR were further included in the multivariate binary logistic regression model. After multivariate adjustment, higher baseline PNI (OR = 1.377, 95% CI: 1.067–1.777, *p* = 0.014) and ALB (OR = 1.840, 95% CI: 1.231–2.749, *p* = 0.003) remained independently associated with a higher probability of achieving disease control in advanced NSCLC patients receiving ICIs. In contrast, BMI did not retain statistical significance in the multivariate model (OR = 0.836, 95% CI: 0.670–1.042, *p* = 0.110), suggesting that its predictive contribution to DCR may be less robust after adjustment for PNI and ALB (Table 4).

**Table 3:** Univariate binary logistic regression analysis of ORR and DCR in patients receiving ICIs.

Variable	B	SE	Wald *χ*^2^	*p* value	OR	95% CI
ORR						
PNI	0.188	0.080	5.591	0.018	1.207	1.033–1.411
ALB	0.172	0.073	5.530	0.019	1.188	1.029–1.371
BMI	0.123	0.048	6.624	0.010	1.131	1.030–1.243
DCR						
PNI	0.281	0.089	10.054	0.002	1.325	1.113–1.577
ALB	0.406	0.098	17.240	<0.001	1.500	1.239–1.817
BMI	0.162	0.059	7.489	0.006	1.176	1.047–1.321

ORR, objective response rate; DCR, disease control rate; PNI, prognostic nutritional index; ALB, albumin; BMI, body mass index; B, regression coefficient; SE, standard error; OR, odds ratio; CI, confidence interval.

**Table 4:** Multivariate binary logistic regression analysis of DCR in patients receiving ICIs.

Variable	B	SE	Wald *χ*^2^	*p* value	OR	95% CI
PNI	0.320	0.130	6.058	0.014	1.377	1.067–1.777
ALB	0.610	0.205	8.853	0.003	1.840	1.231–2.749
BMI	−0.180	0.112	2.551	0.110	0.836	0.670–1.042

DCR, disease control rate; ICIs, immune checkpoint inhibitors; PNI, prognostic nutritional index; ALB, albumin; BMI, body mass index; B, regression coefficient; SE, standard error; OR, odds ratio; CI, confidence interval.

### 3.3 Kaplan–Meier assessment of long-term survival outcomes and independent prognostic factor analysis

**Figure 1:**
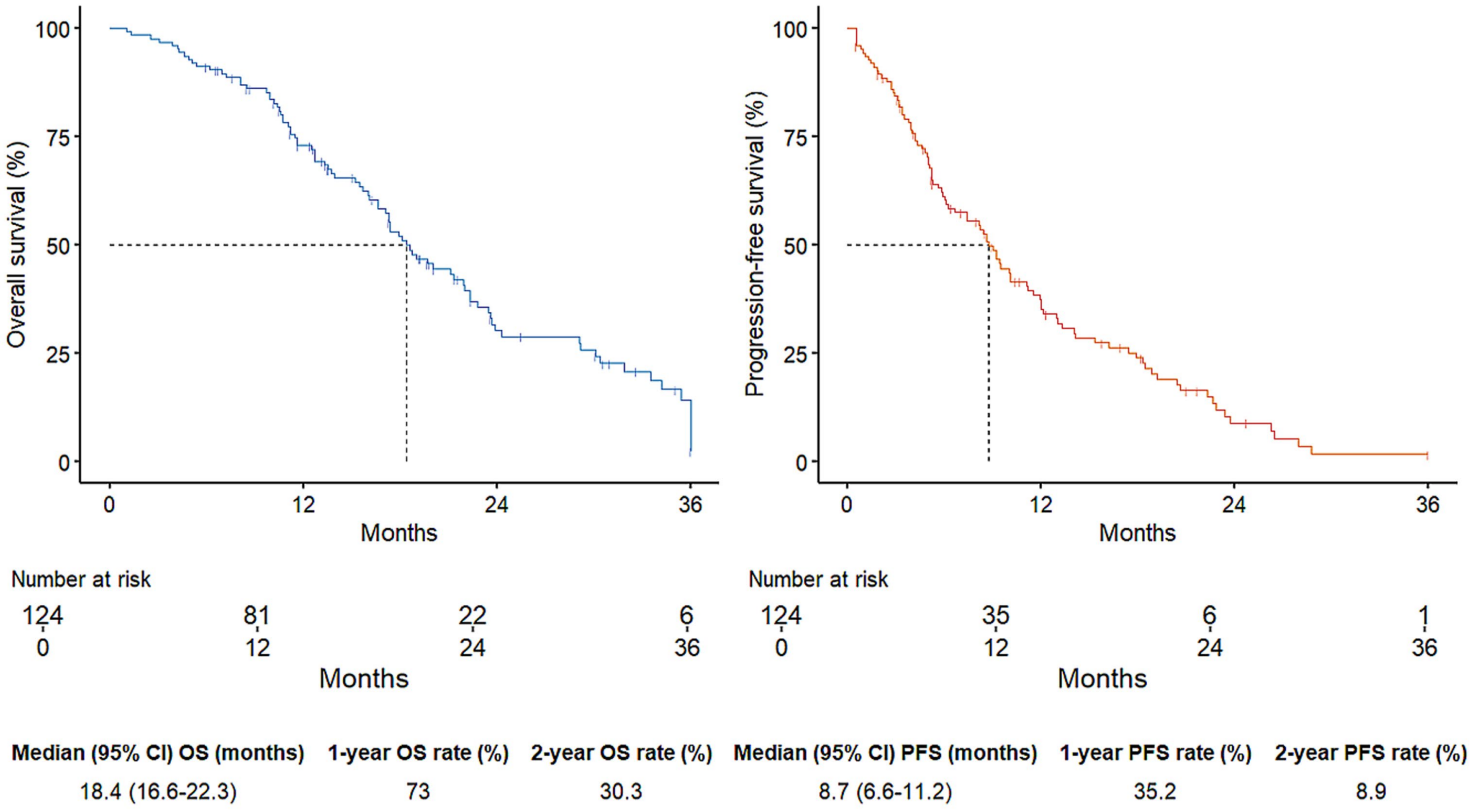
Kaplan–Meier curves for OS and PFS of the entire patient cohort.

**Figure 2:**
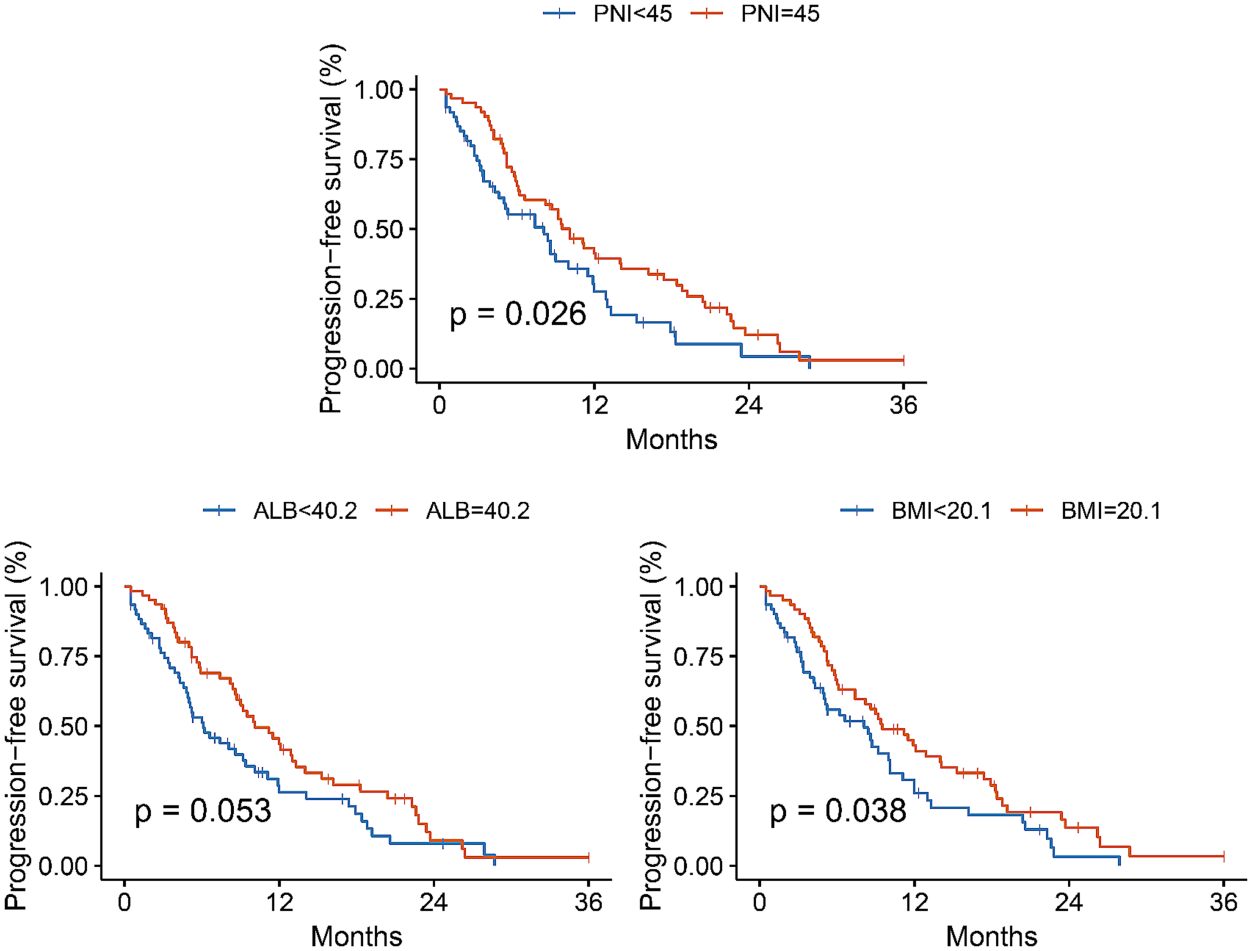
Kaplan–Meier survival curves for PFS stratified by nutritional indicator groups (PNI, ALB, BMI).

**Figure 3:**
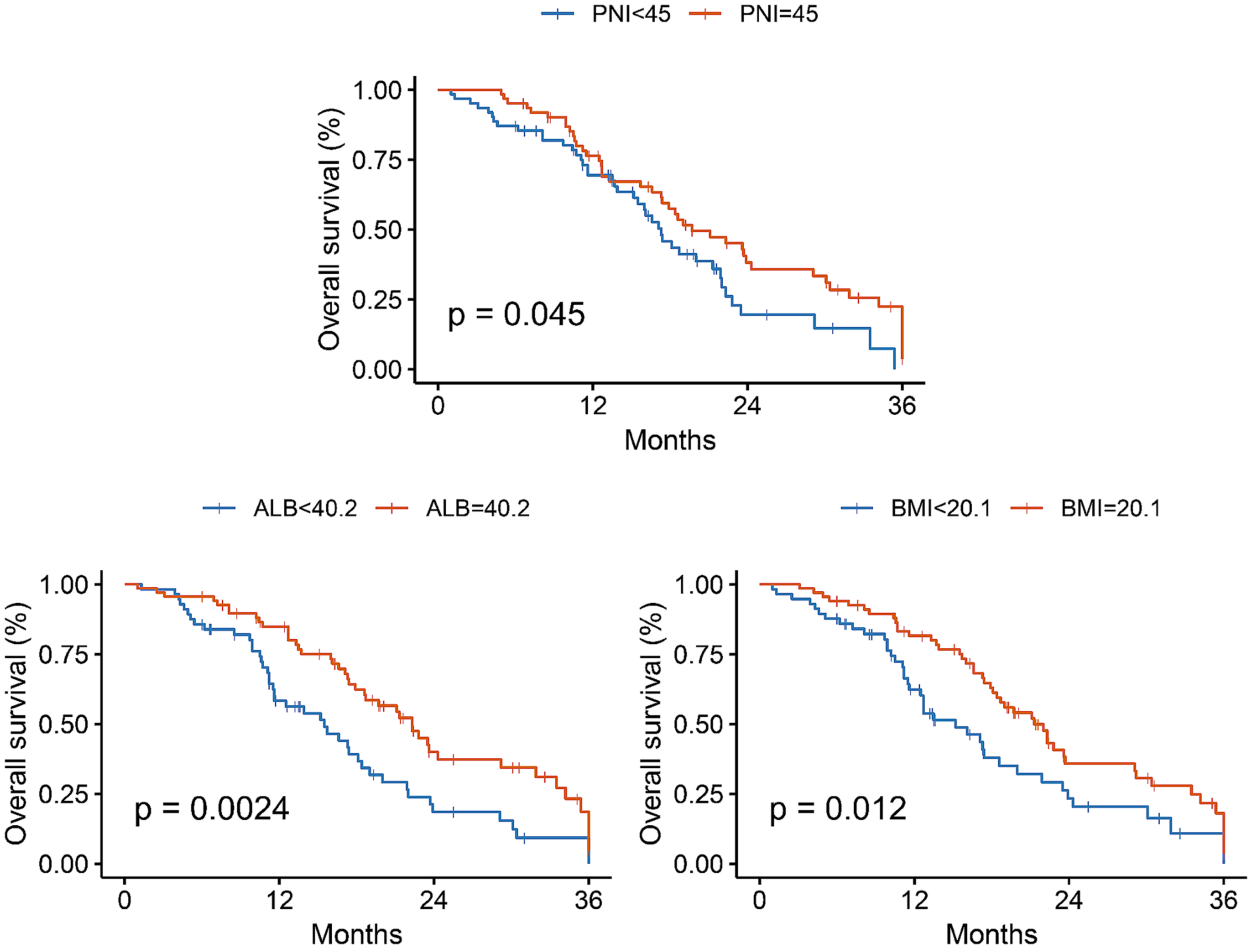
Kaplan–Meier survival curves for OS stratified by nutritional indicator groups (PNI, ALB, BMI).

Kaplan–Meier survival curves showed a median overall survival (OS) of 18.4 months for the entire cohort, with 1-year and 2-year OS rates of 73.0 and 30.3%, respectively; median progression-free survival (PFS) was 8.7 months, with 1-year and 2-year PFS rates of 35.2 and 8.9%, respectively (Figure 1). The Kaplan–Meier survival curves separated early and continued to diverge, showing that both OS and PFS were significantly better in the PNI ≥ 45 group than in the corresponding PNI < 45 group (both *p* < 0.05). Similarly, patients with BMI ≥ 20.1 kg/m^2^ had significantly better OS and PFS than those with low BMI (both *p* < 0.05) (Figures 2, 3). Univariate Cox proportional hazards regression analysis, which is suited for time-to-event survival outcomes, showed that higher baseline PNI and ALB levels were significantly associated with longer PFS and OS. Specifically, PNI was significantly associated with improved PFS (HR = 0.872, 95% CI: 0.785–0.970, *p* = 0.0113) and OS (HR = 0.886, 95% CI: 0.801–0.980, *p* = 0.0191). Similarly, ALB was significantly associated with improved PFS (HR = 0.939, 95% CI: 0.899–0.980, *p* = 0.00407) and OS (HR = 0.927, 95% CI: 0.886–0.971, *p* = 0.00116). In contrast, BMI was not significantly associated with either PFS or OS in univariate Cox analysis (Table 5). After multivariate adjustment, higher baseline PNI remained independently associated with favorable PFS (HR = 0.894, 95% CI: 0.826–0.968, *p* = 0.00591) and OS (HR = 0.916, 95% CI: 0.847–0.989, *p* = 0.0256). Higher ALB was also independently associated with improved PFS (HR = 0.973, 95% CI: 0.947–0.999, *p* = 0.0434) and OS (HR = 0.864, 95% CI: 0.779–0.957, *p* = 0.00532) (Table 6). These findings suggest that baseline PNI and ALB may serve as independent protective prognostic indicators for long-term survival outcomes in patients with advanced NSCLC receiving ICI therapy.

**Table 5:** Univariate Cox proportional hazards regression analysis of PFS and OS in patients receiving ICIs.

Variable	HR	95% CI	*p* value
PFS			
PNI	0.872	0.785–0.97	0.0113
ALB	0.939	0.899–0.98	0.00407
BMI	0.992	0.955–1.03	0.683
OS			
PNI	0.886	0.801–0.98	0.0191
ALB	0.927	0.886–0.971	0.00116
BMI	0.939	0.842–1.047	0.254

PFS, progression-free survival; OS, overall survival; ICIs, immune checkpoint inhibitors; PNI, prognostic nutritional index; ALB, albumin; BMI, body mass index; HR, hazard ratio; CI, confidence interval.

**Table 6:** Multivariate Cox proportional hazards regression analysis of PFS and OS in patients receiving ICIs.

Variable	HR	95% CI	*p* value
PFS			
PNI	0.894	0.826–0.968	0.00591
ALB	0.973	0.947–0.999	0.0434
BMI	0.873	0.759–1.000	0.0569
OS			
PNI	0.916	0.847–0.989	0.0256
ALB	0.864	0.779–0.957	0.00532
BMI	0.900	0.797–1.020	0.0919

PFS, progression-free survival; OS, overall survival; ICIs, immune checkpoint inhibitors; PNI, prognostic nutritional index; ALB, albumin; BMI, body mass index; HR, hazard ratio; CI, confidence interval.

### 3.4 Evaluation of the clinical predictive performance of the combined nutritional Indicator model for survival outcomes

**Figure 4:**
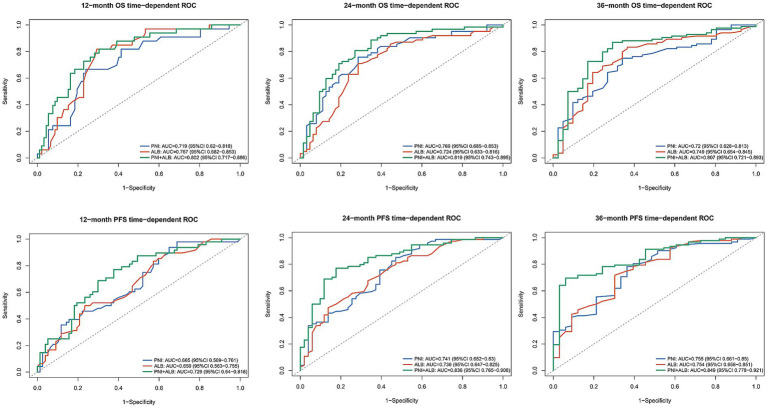
Time-dependent receiver operating characteristic (ROC) curves for the prognostic performance of PNI, ALB, and the combined PNI + ALB model in predicting OS and PFS at 12, 24, and 36 months. The combined model demonstrated consistently higher area under the curve (AUC) values compared with PNI or ALB alone across all time points, indicating improved discriminatory ability.

**Figure 5:**
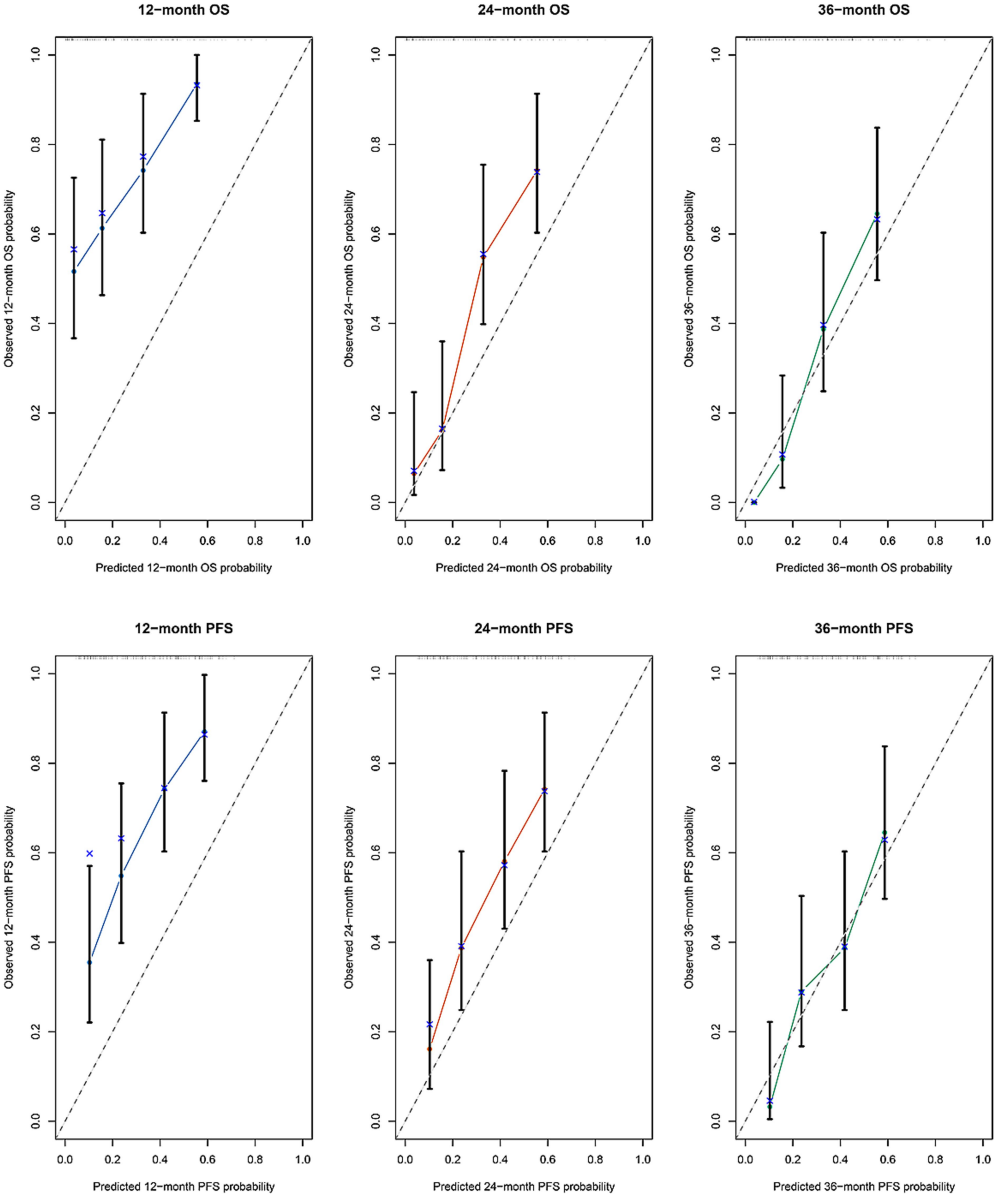
Calibration curves of the combined PNI + ALB model for predicting OS and PFS at 12, 24, and 36 months. The curves were generated using bootstrap resampling and illustrate the agreement between predicted and observed survival probabilities. The 45-degree diagonal line represents the ideal reference line, and closer alignment between the calibration curve and the reference line indicates better predictive accuracy.

Time-dependent receiver operating characteristic (ROC) curve analysis was performed to evaluate the predictive performance of PNI, ALB, and the combined PNI + ALB model for OS and PFS at 12, 24, and 36 months. For OS prediction, the combined model consistently demonstrated superior discriminative ability compared with PNI or ALB alone across all time points. At 12 months, the AUC values were 0.719 for PNI (95% CI: 0.620–0.818), 0.767 for ALB (95% CI: 0.682–0.853), and 0.802 for the combined PNI + ALB model (95% CI: 0.717–0.886). At 24 months, the corresponding AUC values were 0.769 for PNI (95% CI: 0.685–0.853), 0.724 for ALB (95% CI: 0.633–0.816), and 0.819 for the combined model (95% CI: 0.743–0.895). At 36 months, the combined model maintained the highest predictive performance, with AUC values of 0.720 for PNI (95% CI: 0.626–0.813), 0.749 for ALB (95% CI: 0.654–0.845), and 0.807 for the combined model (95% CI: 0.721–0.893). Similar findings were observed for PFS prediction. At 12 months, the AUC values were 0.665 for PNI, 0.659 for ALB, and 0.729 for the combined PNI + ALB model. At 24 months, the AUC values were 0.741, 0.736, and 0.836 for PNI, ALB, and the combined model, respectively. At 36 months, the corresponding AUC values were 0.755, 0.754, and 0.849, respectively. These results indicate that the combined PNI + ALB model provided improved prognostic discrimination compared with either single marker, particularly for 24- and 36-month PFS prediction (Figure 4). Calibration curves were constructed using bootstrap resampling to evaluate the agreement between predicted and observed survival probabilities. For OS, the 12-month calibration curve showed a mild deviation from the ideal 45-degree reference line, whereas the 24- and 36-month calibration curves demonstrated better agreement between predicted and observed probabilities. For PFS, the calibration curves also showed acceptable agreement across the 12-, 24-, and 36-month time points, with relatively better calibration at 24 and 36 months. Overall, these findings suggest that the combined PNI + ALB model exhibited favorable discrimination and acceptable calibration for individualized OS and PFS prediction (Figure 5).

## 4 Discussion

This study systematically evaluated the predictive value of baseline routine nutritional and hematological markers—specifically PNI, ALB, BMI, and Hb—for short-term efficacy and long-term survival outcomes in advanced NSCLC patients receiving ICI therapy. Our findings demonstrated that elevated baseline PNI and ALB levels were significant independent protective factors for superior short-term disease control (DCR) as well as prolonged PFS and OS. Although BMI was correlated with prognosis in the univariate analysis, it lost its independent predictive value after multivariate Cox regression adjustment. Furthermore, this study confirmed that the composite prediction model combining PNI and ALB consistently outperformed single markers, exhibiting enhanced prognostic discrimination and clinical utility for long-term survival benefits.

First, our study confirmed that PNI is an independent predictor of efficacy and survival outcomes in advanced NSCLC patients receiving ICI therapy. This finding is highly consistent with multiple previous studies. Shoji et al. (14) first systematically reported the prognostic value of pretreatment PNI in NSCLC patients receiving immunotherapy, finding that patients in the high PNI group had significantly better OS than those in the low PNI group. Subsequently, several multicenter retrospective analyses further validated the robust predictive performance of PNI across different PD-1/PD-L1 inhibitor regimens (17, 21). A 2023 systematic review and meta-analysis aggregated data from multiple international studies and confirmed that low PNI is significantly associated with worse OS and PFS in NSCLC patients receiving immunotherapy, providing high-level evidence for the clinical application of PNI (22). Mechanistically, PNI integrates two core components: serum albumin and peripheral blood lymphocyte count. Albumin levels reflect protein metabolic status and chronic inflammation, while lymphocyte subsets (particularly CD8^+^ T cells) are the effector cell populations mediating anti-tumor immune responses to ICIs. Therefore, PNI essentially quantifies the patient’s immuno-nutritional reserve status before treatment, effectively predicting the body’s ability to respond to immunotherapy (19, 23). In terms of diagnostic capability, PNI has demonstrated promising performance in discriminating ICI responders from non-responders even before treatment initiation. Its strength lies in combining two complementary biological dimensions—systemic protein-energy status and circulating lymphocyte-mediated immune competence—into a single, readily available index, thereby enhancing diagnostic specificity compared with univariate hematology tests. Given its low cost and high reproducibility, PNI is particularly suited to serve as an initial triage tool to identify patients at high risk of primary resistance, enabling clinicians to prioritize resource allocation.

Second, our study confirmed that baseline ALB level has significant independent predictive value for ICI efficacy. This result aligns with findings from several recent studies. Veccia et al. (24) found in a prospective cohort study that serum albumin was an independent prognostic factor for OS in NSCLC patients receiving immunotherapy, with predictive value even surpassing some traditional inflammatory markers. Chen et al. (25) also observed a strong association between ALB and PFS and OS in esophageal cancer patients receiving immunotherapy, further supporting the potential of albumin as a pan-tumor immuno-nutritional marker. From a pathophysiological perspective, hypoalbuminemia is often closely associated with tumor-related chronic systemic inflammation, cachexia tendency, and microvascular leakage (26). Chronic inflammation can lead to excessive release of immunosuppressive cytokines (such as IL-6, TNF-*α*), thereby inhibiting T cell activation and promoting regulatory T cell (Treg) proliferation, impairing the body’s anti-tumor immune response (18). In addition, amino acid metabolic disturbances under malnutrition can affect the metabolic reprogramming of effector T cells, reducing their ability to infiltrate the tumor microenvironment (27). From a diagnostic perspective, baseline ALB demonstrates clear clinical utility in predicting short-term ICI response and long-term survival, with its dichotomization around the median providing excellent risk stratification capability. Albumin’s diagnostic value is further reinforced by its inverse correlation with systemic inflammatory burden; patients presenting with hypoalbuminemia frequently exhibit elevated IL-6/CRP ratios and a myeloid-derived suppressor cell (MDSC)-dominated immunosuppressive phenotype (28), which can be readily recognized as a diagnostic signature of reduced ICI responsiveness. Importantly, unlike tissue-based biomarkers (e.g., PD-L1 or TMB) that require biopsy and advanced sequencing, ALB can be determined from a routine biochemistry panel with turnaround times of hours, making it a highly practical first-line diagnostic complement in the pre-treatment workup algorithm.

Regarding the predictive value of BMI for ICI efficacy, our results show interesting characteristic findings. Higher BMI was associated with better ORR and survival outcomes in univariate analysis, but this association disappeared in the multivariate model. This phenomenon may reflect the limitations of BMI as a single nutritional indicator. Numerous studies suggest that there may be a complex nonlinear relationship between BMI and survival outcomes in cancer patients—the so-called obesity paradox (29). A recent systematic review by Golban et al. (30) pointed out that a simple BMI indicator fails to distinguish between adipose tissue and skeletal muscle mass, and the latter (particularly the skeletal muscle index) has been shown to be more closely associated with immunotherapy efficacy. Magri et al. (31) found that cancer-related cachexia-induced muscle loss more accurately reflects metabolic disturbances and immunosuppressive status than simple low BMI. Therefore, BMI losing its independent predictive value in multivariate analysis may be related to its inability to fully capture the patient’s true nutritional reserve and body composition characteristics, suggesting that future studies should consider introducing skeletal muscle mass measured by CT or DXA as a more precise nutritional assessment indicator.

A notable innovation of this study lies in the construction and validation of a combined peripheral blood prognostic model integrating PNI and ALB. Time-dependent receiver operating characteristic curve analysis revealed that this combined model demonstrated consistently superior discriminative ability for long-term survival outcomes compared with either single marker across all time points. Specifically, for OS prediction, the combined model achieved robust 12-, 24-, and 36-month AUC values of 0.802, 0.819, and 0.807, respectively, consistently maintaining a high level of discrimination above 0.80. This finding holds profound clinical translational implications, echoing the conclusions of Lei et al. (32), who similarly suggested that the predictive performance of inflammation-nutrition composite indicators is superior to single markers in a stage IV NSCLC population. Pathophysiologically, PNI effectively quantifies the patient’s immuno-nutritional reserve by combining serum albumin levels (reflecting protein metabolic status and chronic inflammation) with total lymphocyte counts (representing the effector cell populations mediating anti-tumor immune responses). Concurrently, baseline ALB serves as a core diagnostic index inversely correlated with systemic inflammatory burden and tumor-related metabolic disturbances. Combining these two complementary biological dimensions allows for a more comprehensive, multidimensional characterization of the patient’s baseline nutrition-immune network. More importantly, both PNI and ALB are standard parameters derived from routine biochemistry panels and complete blood counts, offering the distinct advantages of being non-invasive, low-cost, and highly reproducible. Consequently, this combined model is highly practical and feasible for widespread clinical implementation, even in resource-limited primary healthcare settings.

However, this study has several limitations that need to be addressed in future research. First, this was a retrospective study with a single-center data source and limited sample size (*n* = 124), which may introduce selection bias. Future multicenter, large-sample prospective cohort studies are needed to further validate the external applicability of our findings. Second, the cut-off values for nutritional markers in this study were determined based on the median values of our specific cohort (PNI = 45, ALB = 40.2 g/L, Hb = 114 g/L). While this approach ensures balanced sample sizes between subgroups, it is highly data-dependent and heavily conditioned by our relatively small sample size (*N* = 124). Consequently, these sample-specific cut-offs inherently limit the generalizability and external extrapolation of our findings to broader clinical populations. Our specific thresholds may exhibit altered predictive sensitivity and specificity when applied to other institutions with different baseline nutritional landscapes. To overcome this lack of generalizability, future large-scale, multicenter studies should employ unbiased optimization tools, such as X-tile software or time-dependent ROC curve analysis, to establish standardized and universally applicable cut-off values (15, 33). Third, this study did not include other important biomarkers that may affect ICI efficacy (such as PD-L1 expression, TMB, and peripheral blood inflammatory markers like NLR and PLR) (34, 35). Future studies should consider constructing multidimensional integrated prediction models encompassing molecular markers, nutritional indicators, and inflammatory markers. Fourth, due to the retrospective design, we were unable to assess dynamic changes in nutritional markers during treatment. Several studies have shown that dynamic changes in PNI and ALB during ICI therapy also have prognostic predictive value (36, 37). Future prospective studies should incorporate dynamic monitoring into their design to more comprehensively reveal the temporal relationship between nutritional status and immunotherapy response. Fifth, our cohort was predominantly adenocarcinoma cell carcinoma (71.8%), with a relatively low proportion of squamous; different pathological subtypes may have different nutrition-immune characteristics (38). Although establishing an independent external validation cohort was logistically challenging for the present study, we acknowledge that the lack of external validation remains a significant limitation, which further restricts the immediate generalizability and clinical extrapolation of our predictive models. Future prospective, multi-center studies with independent validation cohorts are strictly required to verify the reproducibility and universal clinical utility of these biomarkers.

In conclusion, this study confirms that baseline PNI and ALB are independent predictors of short-term efficacy and long-term survival outcomes in advanced NSCLC patients receiving ICI therapy, and the combined PNI and ALB model significantly outperforms single indicators in predictive performance. These findings provide evidence-based support for optimizing individualized immunotherapy strategies for advanced NSCLC and have important clinical translational value. Future multicenter prospective studies will further validate and expand the conclusions of this study.

## Data Availability

The original contributions presented in the study are included in the article/supplementary material, further inquiries can be directed to the corresponding author/s.
